# Survival rates of cancer patients with and without rheumatic disease: a retrospective cohort analysis

**DOI:** 10.1186/s12885-016-2444-5

**Published:** 2016-07-04

**Authors:** Jin Kyun Park, Ji Ae Yang, Eun Young Ahn, Sung Hae Chang, Yeong Wook Song, Jeffrey R. Curtis, Eun Bong Lee

**Affiliations:** Division of Rheumatology, Department of Internal Medicine, Seoul National University College of Medicine, 101, Daehak-ro, Jongno-gu, Seoul, 03080 Korea; Division of Rheumatology, Department of Internal Medicine, Soonchunhyang University Cheonan Hospital, Cheonan, South Korea; Division of Clinical Immunology & Rheumatology, University of Alabama at Birmingham, Birmingham, AL USA

**Keywords:** Rheumatic diseases, Cancer, Staging, Mortality, Survival

## Abstract

**Background:**

To compare the outcomes of gastric, colon, lung, and breast cancer patients with and without rheumatic diseases (RD).

**Methods:**

This retrospective study compared the cancer survival rates of a cohort of 122 cancer patients with rheumatoid arthritis (RA), systemic lupus erythematosus (SLE), dermatomyositis/polymyositis (DM/PM), or systemic sclerosis with that of a cohort of 366 age-, sex-, and, cancer type-matched patients without RD who received medical care from 2000 to 2014. Staging, comorbidities, and functional status were ascertained. Survival was compared using the Kaplan-Meier method. Relative risk of death was estimated as a hazard ratio (HR) using Cox regression analysis.

**Results:**

The mean age of the RD patients at the time of cancer diagnosis was 58.7 ± 11.5 years. The overall survival rate of gastric cancer patients did not differ between the cohorts. The survival of lung or breast cancer was worse in patients with RA or DM/PM than in those without RD (all, *p* < 0.05). After adjusting for cancer stage, comorbidity index, performance status and age at the time of cancer diagnosis (as well as interstitial lung disease for lung cancer group), the mortality rate among lung cancer patients with RA was significantly higher (HR, 1.81; 95 % CI, 1.03–3.18) than that of lung cancer patients without RD, whereas SSc was associated with decreased mortality of lung cancer (HR, 0.16; 95 % CI, 0.04–0.58). DM/PM were associated with increased mortality of breast cancer patients (HR, 297.39; 95 % CI, 4.24–20842.33).

**Conclusions:**

RA and DM/PM seemed to be associated with a higher mortality in patients with lung or breast cancers, whereas SSc seemed to be associated with decreased mortality in patients with lung cancer. It is warranted to explore the survival effect of tailored cancer treatments according to specific RD.

**Electronic supplementary material:**

The online version of this article (doi:10.1186/s12885-016-2444-5) contains supplementary material, which is available to authorized users.

## Background

Rheumatic disease (RD) is characterized by chronic systemic inflammation involving multiple organs. Unopposed inflammation and the production of cytokines, growth hormones, and toxic reactive oxygen species may promote the progression of precancerous cells into a clinically significant cancer [1]. Impaired immune surveillance associated with underlying RD and long-term immunosuppressive therapy might not only promote the survival and proliferation of cancer cells, but also increase the risk of infection, a main cause of death during cancer treatment [2–4]. In short, cancer patients with RD may have a worse prognosis than those without RD. Conversely, cytotoxic autoimmune cells in patients with RD might eliminate cancer cells better. Also, the prolonged use of non-steroidal anti-inflammatory drugs (NSAIDs) and antimetabolites such as methotrexate might inhibit proliferative stimuli, thereby inhibiting cancer development and progression [5, 6]. Furthermore, the close medical monitoring received by RD patients during longitudinal follow up might lead to early detection of cancers and more prompt treatment, ultimately resulting in a better outcome [7, 8].

Although a solid body of evidence supports a close association between RD and incident cancers, data regarding outcomes for RD patients who develop incident cancer are scarce and often conflicting [9, 10]. We previously reported that the cancer outcome in patients with Takayasu arteritis was excellent, suggesting that the underlying autoimmune disease might not negatively influence cancer outcome, at least for certain RDs [11]. Here, we aimed to investigate whether cancer patients with specific RDs have a worse outcome than those without RDs by comparing the survival of RD patients with four common cancers (i.e., gastric, colon, lung, and breast) with that of matched cancer patients without RDs.

## Methods

### Patients

The medical records of patients with rheumatoid arthritis (RA), systemic lupus erythematosus (SLE), systemic sclerosis (SSc), and dermatomyositis/polymyositis (DM/PM) that received longitudinal clinical care at Seoul National University Hospital from January 2000 to April 2014 were retrospectively reviewed, and those that had developed a gastric, colon, lung, or breast cancer after their RD diagnosis were enrolled in the study (these patients comprised the RD-exposed cancer cohort). The patients in whom cancer was diagnosed before RD were excluded, since the aim of the study was to investigate the effects of RD on the cancer outcome. RA, SLE, SSc and PM/DM were diagnosed according to the 1987 revised classification criteria of the American College of Rheumatology (ACR) for RA, the 1997 ACR classification criteria for SLE, the 1980 preliminary ACR classification criteria for SSc, and the Bohan and Peter criteria for PM or DM, respectively [12–15]. The non-RD-exposed cancer cohort comprised 366 age-, sex-, and cancer site-matched patients without RD who were diagnosed with cancer in the same year as the RD patients and who received medical care at the same hospital. These patients were randomly selected from the medical record archive so as to achieve a matching ratio of 1 to 3 for each RD. The study was approved by the Institutional Review Board of the Seoul National University Hospital. The need for patient consent was waived by the Review Board as the study involved minimal risk and its retrospective nature meant that no identifiable information was used.

### Cancer assessment

A complete data set for each cancer, including histology, stage, and treatment, was obtained. All cancer cases were diagnosed histopathologically and confirmed by biopsy. Cancers were staged according to the American Joint Committee on Cancer (AJCC) 7th staging system. The Eastern Cooperative Oncology Group performance status (ECOG-PS) and the Charlson comorbidity index at the time of cancer diagnosis were estimated after reviewing electronic medical records. An ECOG-PS of 0 indicates that the patient is asymptomatic and a score of 1 indicates that patient is restricted in terms of strenuous activity but is able to ambulate and carry out light work; however, an ECOG-PS ≥2 indicates a significant limitation in performance status [16]. The Charlson comorbidity index predicts 10-year mortality for a patient who may have a range of 22 comorbid conditions, including cardiovascular disease and lung disease [17]. The index sums the assigned scores for each comorbid condition, e.g., connective tissue disease is assigned a score of 1, while a malignant tumor is assigned a score of 6. In this study, the scores for connective tissue disease and cancer were omitted from the Charlson comorbidity index.

### Statistical analysis

Student t-tests or analysis of variance (ANOVA) were used to compare continuous variables between groups and the Chi-square or Fisher’s exact test were used to compare categorical variables. Survival curves were generated using the Kaplan-Meier method. Differences in survival were compared using the log-ranks test. Follow up began at the time of cancer diagnosis and was censored at the time of death or on the last day on which survival status was followed up, whichever came first. The survival status of the all patients was ascertained using the National Death Register of Statistics Korea (www.kostat.go.kr). The relative risk of death was estimated using Cox’s proportional hazard ratio (HR) for survival by adjusting for stage, ECOG performance status, Charlson comorbidity index, and age in years at the time of cancer diagnosis. HR for lung cancer mortality was adjusted for the interstitial lung disease (ILD) status as well. *P* ≤0.05 was considered significant. All analyses were performed using IBM SPSS (statistics version 19.0, Chicago, IL, USA).

## Results

### Demographic characteristics of RD patients with cancer

During the follow-up period, 122 RD patients with one of the four selected types of incident cancer were identified. RA was the most common RD (80 patients; 65.6 %), followed by DM/PM (16 patients; 13.1 %), SSc (13 patients; 10.7 %), and SLE (13 patients; 10.7 %). The mean age at RD diagnosis was 52.4 ± 13.5 years (Table 1). Except for the DM/PM group, the number of females was higher than that of males. There were 28 cases of gastric cancer (23.0 %), 23 cases of colon cancer (18.9 %), 44 cases of lung cancer (36.1 %), and 27 cases of breast cancer (22.1 %). The mean age of the RD patients at the time of cancer diagnosis was 58.7 ± 11.5 years. The mean time from RD diagnosis to cancer diagnosis was 5.8 [range: 0, 36.3] years. Notably, the mean time between DM/PM and cancer diagnoses was 2.2 [range: 0, 8.4] years. The time between the breast cancer and DM/PM diagnosis was relatively longer as compared to gastric, colon and lung cancer (Additional file 1: Table S1).

**Table 1:** Characteristics of 122 patients with rheumatic diseases and 366 cancer-matched controls without RD

	RA (*n* = 80)	SLE (*n* = 13)	SSc (*n* = 13)	DM/PM (*n* = 16)	All RD (*n* = 122)	Non-RD (*n* = 366)
Female, *n* (%)	59 (73.8)	13 (100.0)	10 (76.9)	6 (37.5)	88 (72.1)	264 (72.1)
Age at RD dx, yrs,	54.7 ± 12.8	42.4 ± 12.9	45.6 ± 16.1	58.2 ± 9.9	52.4 ± 13.5	N/A
Age at cancer dx, yrs,	60.2 ± 11.3	52.2 ± 9.7	54.2 ± 13.9	59.7 ± 9.3	58.7 ± 11.5	58.6 ± 11.6
Follow-up duration, yrs	14.4 ± 12.0	17.3 ± 10.2	10.7 ± 7.1	13.0 ± 11.2	12.8 ± 9.7	12.8 ± 9.7
Cancer type, *n* (%)						
Stomach	18 (22.2)	4 (30.8)	2 (15.4)	4 (25.0)	28 (23.0)	83 (22.7)
Colon	16 (20.0)	3 (23.1)	1 (7.7)	3 (18.8)	23 (18.9)	69 (18.9)
Lung	28 (35.0)	0 (0)	10 (76.9)	6 (37.5)	44 (36.1)	133 (36.3)
Breast	18 (22.5)	6 (46.2)	0 (0.0)	3 (18.8)	27 (22.1)	81 (22.1)
Interstitial lung disease, *n* (%)	6 (7.5)	1 (7.7)	7 (53.8)	3 (18.8)	17 (13.9)	3 (0.8)
Treatment of RD, *n* (%)						
Methotrexate	53 (66.3)	1 (7.7)	0 (0)	5 (31.3)	59 (48.4)	N/A
Other DMARDs	57 (71.3)	6 (46.2)	2 (15.4)	6 (37.5)	71 (58.2)	N/A
Corticosteroids	48 (60.0)	7 (58.3)	4 (30.8)	13 (81.3)	72 (59.0)	N/A
TNF inhibitors	4 (5.0)	0 (0)	0 (0)	0 (0)	4 (3.3)	N/A
Others^a^	5 (6.3)	4 (30.8)	2 (15.4)	4 (25.0)	15(12.3)	N/A

Data are expressed as the mean ± SD or as number (%)

*Dx* diagnosis, *DMARD* disease modifying antirheumatic drug, *DM/PM* dermatomyositis/polymyositis, *N* number, *N/A* not applicable, *RA* rheumatoid arthritis, *RD* rheumatic disease, *SLE* systemic lupus erythematosus, *SSc* systemic sclerosis, *TNF* tumor necrosis factor, *Yrs* years

^a^Others include tocilizumab, rituximab, abatacept, bucillamine, and cyclosporine

### Characteristics of RD patients with cancer

The ECOG performance score for RD patients with colon cancer was better than that for their matched non-RD counterparts whereas that for RD patients with lung cancer was worse (Table 2, upper row).

**Table 2:** Baseline cancer-related characteristics of the 122 RD patients with cancer and the 366 age-, sex-, and cancer-matched controls without RD

	Gastric			Colon			Lung			Breast		
	RD (*n* = 28)	Non-RD (*n* = 83)	*p*	RD (*n* = 23)	Non-RD (*n* = 69)	*p*	RD (*n* = 44)	Non-RD (*n* = 133)	*p*	RD (*n* = 27)	Non-RD (*n* = 81)	*p*
ECOG^c^												
≤1	28 (100.0)	81 (97.6)	1.000^b^	22 (95.7)	45 (65.2)	0.006b	34 (77.3)	119 (89.5)	0.041^a^	27 (100.0)	81 (100.0)	1.000^b^
≥2	0 (0)	2 (2.4)	1.000^b^	1 (4.3)	24 (34.8)	0.006b	10 (22.7)	14 (10.5)	0.041 ^a^	0 (0)	0 (0)	1.000
Charlson^d^												
0	16 (57.1)	54 (65.1)	0.501^b^	8 (34.8)	46 (66.7)	0.025^b^	22 (50.0)	90 (67.7)	0.047^a^	18 (66.7)	65 (80.2)	0.188^b^
1	5 (17.9)	17 (20.5)	1.000^b^	8 (34.8)	12 (17.4)	0.090^b^	12 (27.3)	22 (16.5)	0.127b	4 (14.8)	11 (13.6)	1.000^b^
≥2	7 (25.0)	12 (14.5)	0.246^a^	7 (30.4)	11 (15.9)	0.141^b^	11 (22.7)	21 (15.8)	0.180b	5 (18.5)	5 (6.2)	0.117^b^
Stage												
I	13 (46.4)	41 (49.4)	0.830^b^	7 (30.4)	12 (17.4)	0.234^b^	14 (31.8)	27 (20.3)	0.149^b^	14 (51.9)	24 (29.6)	0.036^a^
II	6 (21.4)	15 (18.1)	0.781^b^	5 (21.7)	14 (20.3)	1.000^b^	5 (11.4)	77 (57.9)	<0.001 ^b^	10 (37.0)	44 (54.3)	0.120^a^
III	1 (3.6)	6 (7.2)	0.677^b^	4 (17.4)	23 (33.3)	0.190^b^	6 (13.6)	15 (11.3)	0.788 ^b^	1 (3.7)	6 (7.4)	0.678^b^
IV	7 (25.0)	20 (24.1)	1.000^b^	7 (30.4)	18 (26.1)	0.788^b^	16 (36.4)	14 (10.5)	<0.001 ^b^	2 (7.4)	2 (2.5)	0.260^b^
NA	1 (3.6)	1 (1.2)	0.443^b^	0 (0)	2 (2.9)	1.000^b^	3 (6.8)	0 (0)	0.015 ^b^	0 (0)	5 (6.2)	0.328^b^

Data are expressed as number (%)

*F* female, *NA* not applicable, *RD* rheumatic disease

^a^Chi-square test, ^b^Fisher’s exact test

^c^Eastern Cooperative Oncology Group (ECOG) performance status

^d^The Charlson comorbidity index was adjusted for connective tissue disease and cancer

Although the proportion of RD patients with gastric, colon, and lung cancer with a Charlson comorbidity score ≥1 was greater than that of matched cancer patients without RD, the difference was significant for RD patients with colon cancer (65.2 % for patients with RD *vs.* 33.3 % for patients without RD; *p* = 0.025) and those with lung cancer (50.0 % for patients with RD *vs.* 32.3 % for patients without RD; *p* = 0.047) (Table 2, middle row).

Significantly more stage 1 breast cancers were detected in RD patients than in their matched counterparts without RD (51.9 % vs. 29.6 %, respectively; *p* = 0.036). Stage IV lung cancer was more prevalent and stage II less prevalent in RD patients than in their matched counterparts without RD (36.4 % *vs.* 10.5 %; *p* < 0.001 for stage IV and 11.4 % *vs.* 57.9 %; *p* < 0.001 for stage II) (Table 2, bottom rows). Staging of gastric and colon cancers did not differ between the two groups.

### Cancer mortality in RD patients versus non-RD patients

During the follow-up period, 45 (36.9 %) of the 122 patients with RD died. One SSc patient with colon cancer died due to progression of SSc-associated interstitial lung disease (ILD). One RA patient with breast cancer died of congestive heart failure (*n* = 1) and another of old age (*n* = 1). The remaining 42 (93.3 %) of 45 deaths occurred due to the cancer progression or complications during cancer treatment (Table 3).

**Table 3:** Cause of death of 45 patients with rheumatic disease and cancer

	RD	Cancer	Cause of death	Stage	ECOG^a^	Comorbidity^b^
1	DM/PM	Breast	Cancer progression	2	1	1
2	DM/PM	Gastric	Cancer progression	4	0	0
3	DM/PM	Lung	Cancer progression	1	0	0
4	DM/PM	Lung	Cancer progression	1	0	1
5	DM/PM	Lung	Cancer progression	2	2	1
6	DM/PM	Lung	Cancer progression	2	2	0
7	DM/PM	Lung	Cancer progression	3	2	0
8	DM/PM	Lung	Cancer progression	NA	0	0
9	RA	Breast	Cancer progression	2	0	2
10	RA	Breast	Cancer progression	4	1	0
11	RA	Breast	Congestive Heart failure	1	0	1
12	RA	Breast	Old age	2	0	2
13	RA	Colon	Cancer progression	1	1	1
14	RA	Colon	Cancer progression	3	2	2
15	RA	Colon	Cancer progression	4	1	2
16	RA	Lung	Cancer progression	1	0	0
17	RA	Lung	Cancer progression	1	0	0
18	RA	Lung	Cancer progression	1	0	0
19	RA	Lung	Cancer progression	2	1	2
20	RA	Lung	Cancer progression	2	0	0
21	RA	Lung	Cancer progression	2	0	1
22	RA	Lung	Cancer progression	3	2	1
23	RA	Lung	Cancer progression	3	0	1
24	RA	Lung	Cancer progression	3	0	1
25	RA	Lung	Cancer progression	3	1	2
26	RA	Lung	Cancer progression	4	1	0
27	RA	Lung	Cancer progression	4	2	0
28	RA	Lung	Cancer progression	4	0	0
29	RA	Lung	Cancer progression	4	1	1
30	RA	Lung	Cancer progression	4	2	2
31	RA	Lung	Cancer progression	4	2	1
32	RA	Lung	Cancer progression	4	1	0
33	RA	Lung	Cancer progression	4	2	1
34	RA	Lung	Cancer progression	4	0	0
35	RA	Lung	Cancer progression	4	1	0
36	RA	Lung	Cancer progression	4	0	2
37	RA	Lung	Cancer progression	NA	1	0
38	SLE	Breast	Cancer progression	4	1	0
39	SLE	Colon	Cancer progression	1	0	2
40	SSc	Colon	ILD aggrevation	3	1	2
41	SSc	Lung	Cancer progression	4	1	2
42	SSc	Lung	Cancer progression	4	2	1
43	SSc	Lung	Cancer progression	4	1	0
44	SSc	Lung	Cancer progression	4	0	0
45	SSc	Lung	Cancer progression	NA	2	2

*F* female, *NA* not available, *RD* rheumatic disease, *DM* dermatomyositis, *PM* polymyositis, *RA* rheumatoid arthritis, *SLE* systemic lupus erythematosus, *SSc* systemic sclerosis

^a^An Eastern Cooperative Oncology Group (ECOG) performance status

^b^The Charlson comorbidity index was adjusted for connective tissue disease and cancer

**Fig. 1:**
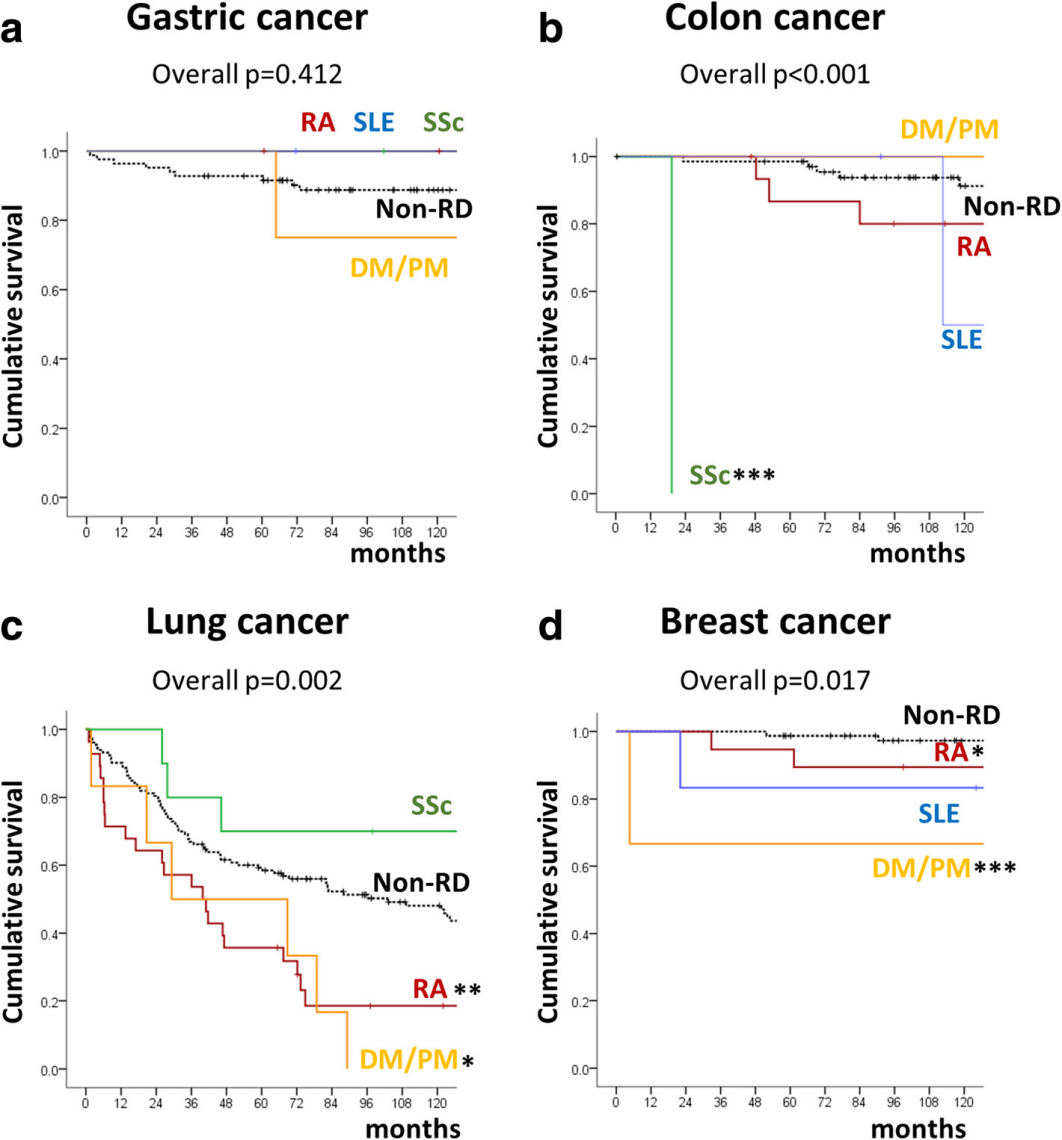
Kaplan-Meier survival curves for cancer patients with gastric cancer (**a**), colon cancer (**b**), lung cancer (**c**) and breast cancer (**d**) were compared with those for matched cancer patients without rheumatic diseases. Tick marks indicate censoring events. The *p* value was calculated using the log rank test. DM, dermatomyositis; ND, not determined; PM, polymyositis; RA, rheumatoid arthritis; RD, rheumatic disease; SLE, systemic lupus erythematosus; SSc, systemic sclerosis.*p < 0.05, **p < 0.01 and ***p < 0.001 compared with the non-RD exposed cancer cohort

The overall survival of patients with gastric cancer did not differ between the two groups (Fig. 1a). Of those with colon cancer, one patient with SSc had a worse outcome than their matched counterparts without RD (Fig. 1b). Mortality was significantly worse for lung and breast cancer patients with RA or DM/PM than for those without RD (Fig. 1c and d).

**Fig. 2:**
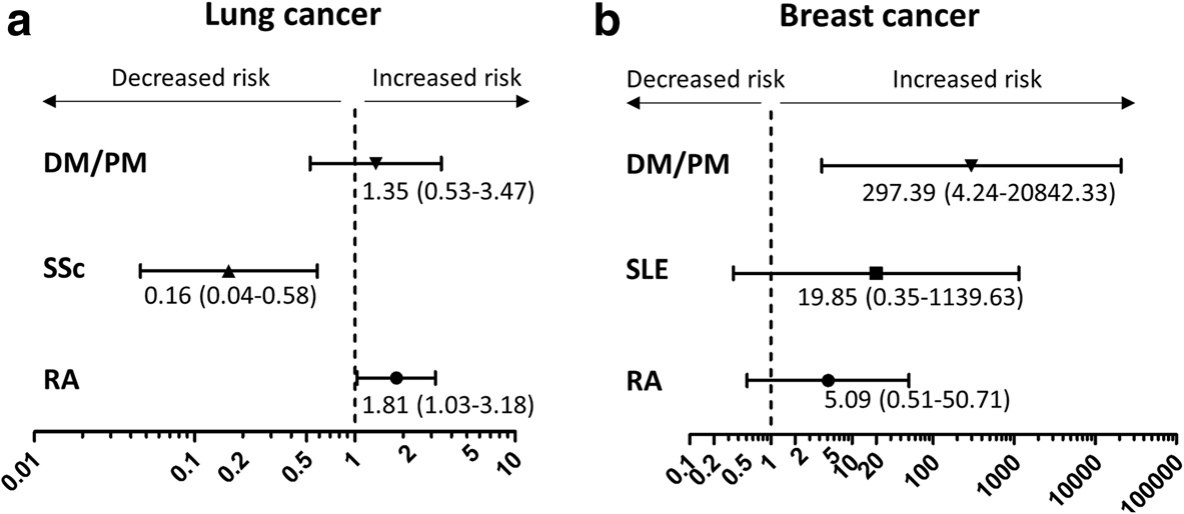
Hazard ratios (HR) and 95 % confidence intervals for overall survival of patients with lung **a** or breast **b** cancer. HR was calculated by adjusting for cancer stage, performance status, comorbidity index, and age at the time of cancer diagnosis. HR for lung cancer was adjusted also for the presence of ILD. ND, not determined; DM, dermatomyositis; PM, polymyositis; RA, rheumatoid arthritis; SLE, systemic lupus erythematosus; SSc, systemic sclerosis

Next, the relative risk of RD with respect to mortality was estimated after adjusting for cancer stage, comorbidity index, performance status, and age at the time of cancer diagnosis. In case of lung cancer, the presence of ILD was also considered for HR calculation. Compared with matched cancer patients without RD, RA was associated with a significant increase in the mortality of lung cancer (HR, 1.81; 95 % CI, 1.03–3.18 (Fig. 2). DM/PM was associated with a 297.39-fold increase in mortality for breast cancer (95 % CI, 4.24–20842.33) patients when compared with matched controls without RD. Strikingly, SSc was associated with decreased mortality for lung cancer (HR 0.16; 95 % CI, 0.04–0.58).

## Discussion

The present study shows that the mortality associated with breast and lung cancer differs markedly depending on the type of RD: patients with DM/PM with lung cancer had a much worse outcome than counterparts without RD, whereas lung cancer patients with SSc had a better outcome than those without RD.

Although all RDs arise due to defective immune response with subsequent chronic inflammation, the difference in immune dysregulation with preferential production of specific cytokines is associated with varying clinical manifestation [18]. Thus, RDs are classified by the main site of involvement; RA and DM/PM involve primarily joints and muscle, respectively, as the main target organ. SSc is characterized by systemic fibrosis which is considered as an overshooting wound healing [19, 20]. Inflammation of SLE is mediated by formation and deposition of immune complexes in many tissues, followed by subsequent complement activation leading to organ damage [21]. Thus, the difference in the immune dysregulation and the associated clinical manifestations in individual RDs might influence their cancer incidence and outcome [10].

Cancer survival depends mainly on cancer stage, comorbidities, and functional status at the time of cancer diagnosis, all of which determine the treatment strategy and outcome [17, 22]. Here, we found that the adjusted Charlson comorbidity index (adjusted for the presence of cancer and connective tissue disease) was worse only for RD patients with colon and lung cancer when compared with matched cancer patients without RD. The ECOG performance status was similar between the RD patients and matched cancer patients without RD (Table 2). This finding suggests that underling RDs might have little influence on performance status and comorbidities in cancer patients.

Since RD patients are followed up more closely during their longitudinal care, we expected that more cancers would be detected at an early stage [7]. However, we found that this was only the case for breast cancer. In Korea, the National Cancer Screening Program (Korean NCSP) recommends that women aged 40 years or over undergo a screening mammogram once every 2 years. The cumulative screening rate for breast cancer in eligible women was reported as high as 77.9 % by 2012 in Korea [23]. One might speculate that their underlying chronic illness makes patients with RD more vigilant about health issues; thus, they are more likely to follow this recommendation, especially since they have better access to health care services. Strikingly, there was no difference in the stage of gastric cancer between RD and non-RD groups. Chronic use of NSAIDs often leads to gastrointestinal discomfort; such patients are more likely to undergo esophagogastroduodenoscopy (EGD), which would detect “incidental” cancers at the early stage [24]. In Korea, the high prevalence of *Helicobacter pylori* and associated gastritis and gastric cancer lower the threshold for EGD, which is readily available at low cost. As for breast cancer, the Korean NCSS offers gastric cancer screening, which includes a double contrast barium upper gastrointestinal series or EGD once every 2 years. In 2012, the cumulative screening rate was reported to be 77.9 % of the eligible people [23]. The lack of difference in gastric cancer staging between the RD-exposed cohort and the non-RD-exposed cohort may be explained by the high screening rate for gastric cancer in the general population in Korea [25]. Advanced lung cancer was detected more often in patients with RD, consistent with a prior report showing that up to 83 % of lung cancers associated with connective tissue disease were detected at stage IV [26]. As RD often affects the lung in the form of ILD, the malignant mass might be hidden by diffuse parenchymal changes until it manifests clinically, leading to delayed cancer detection (Table 2) [27]. Alternatively, the defective immune surveillance associated with RD or immunosuppressive treatment facilitates rapid cancer progression. Of note, the Korean NCSS does not recommend screening for lung cancer. Overall, we found the similar (or at least no better) staging of cancer patients with RD and cancer-matched controls without RD in the present study interesting, and it raises questions as to whether patients with RD might benefit from cancer screening guidelines tailored to their underlying RD. Further studies are needed to compare the cancer screening rates between patients with RD and the general population.

The survival of lung cancer patients with RA or DM/PM was worse than that for SSc patients and those without RD (Fig. 1c). This is striking, since ILD, which was present in 7 (53.8 %) SSc patients in the present study, would negatively affect pulmonary function and, therefore, cancer outcome. As shown in Table 1, ILD develops in a relatively low proportion of RA patients and may be clinically asymptomatic [28]. ILD is rare as in DM/PM patients with cancer, although it is present in ≥60 % DM/PM patients in general [29]. After adjusting for cancer stage, comorbidities, performance status, age at the time of cancer diagnosis, and ILD status, we found that RA *per se* was associated with increased mortality of lung cancer patients by 1.81-fold (95 CI, 1.03–3.18), whereas SSc was associated with decreased mortality of lung cancer (HR, 0.16; 95 % CI, 0.04–0.58). The results presented herein suggest that the additional presence of ILD is not associated with a worse outcome for lung cancer in patients with SSc. It is tempting to speculate that the accumulation of autoimmune cells associated with ILD provides a harsh environment for cancer cells to grow [30, 31].

The overall mortality rate was high for breast cancer patients with RA or DM/PM (Fig. 1d), when compared with the cancer-matched controls without RD (Table 2, bottom row), even though more breast cancers were diagnosed at stage I. After adjusting for cancer stage, comorbidities, and performance status, DM/PM is associated with higher mortality of breast cancer, similar to a prior report [32]. RA also tended to show a higher death rate from breast cancer, suggesting that RA *per* se could have a negative effect on cancer outcome (Fig. 2b). This finding is consistent with those of prior studies showing that patients with RA have a higher cancer-associated mortality than the general population [33–35]. As both RA and breast cancer are diseases that predominantly affect women, changes in female hormone levels might increase the aggressiveness of breast cancer in RA patients [36]. A common defect in the immune response may lead to both autoimmunity and impaired cancer surveillance. Ultimately, the mechanisms by which each RD influences cancer physiology require further investigation. As of now, given the worse prognosis for RD patients with particular cancers, more aggressive cancer treatment tailored to the underlying RD should be the subject of future studies.

The electronic medical records held at our institution allow us to obtain reliable information regarding the demographic and clinical characteristics of the both cancer and RD patients. In addition, the National Death Register enables us to ascertain the survival status of all enrolled patients. However, the study still has several limitations. First, the relatively small cohort size results in estimates with wide CI. Second, the current study design does not allow estimation of the effects of RD therapy on cancer outcome of patients with corresponding RD due to the tight collinearity between RD and RD treatment. This is of particular interest, since the treatment of RD focuses on the immune response, which obviously plays a crucial in anti-tumor immunity. Third, since cancer treatments vary, often at the discretion of the treating physician, it was difficult to thoroughly analyze the effects of cancer treatment on the survival due to relatively small number of the patients. However, we found that cancer patients with RD and those without RD were treated with comparable aggressiveness except for DM/PM patients with lung cancer; none (0 %) of the 6 lung cancer patients with DM/PM underwent a surgery as compared to the 58.6 % in the lung cancer patients without RD (Additional file 1: Table S2). These limitations should be addressed in a larger prospective study. Last but not the least, the survival analyses were adjusted for Charles comorbidity index and ECOG, although both index were not specifically designed to reflect the functional status of RDs. Retrospective nature of this study did not allow us to collect RD-specific functional status.

## Conclusions

In conclusion, lung and breast cancer patients with RA or DM/PM seemed to be associated with worse survival than non-RD counterparts. Further studies will be needed to address whether cancer treatments tailored to specific RD can improve survival.

## Abbreviations

ACR, American College of Rheumatology; AJCC, American Joint Committee on Cancer; ANOVA, analysis of variance; DM, dermatomyositis; ECOG-PS, Eastern Cooperative Oncology Group performance status; EGD, esophagogastroduodenoscopy; HR, hazard ratio; ILD, interstitial lung disease; NCSP, National Cancer Screening Program; NSAIDs, non-steroidal anti-inflammatory drugs; PM, polymyositis; RA, rheumatoid arthritis; RD, rheumatic diseases; SLE, systemic lupus erythematosus; SSc, systemic sclerosis

## Additional file

Additional file 1: **Supplementary material.** **Table S1.** Interval between rheumatic disease and cancer diagnosis in years. **Table S2.** Cancer treatment according to rheumatic diseases. (DOCX 26 kb)
