# Supplementary material for: Survival rates of cancer patients with and without rheumatic disease: a retrospective cohort analysis
Source: BMC Cancer. 2016 Jul 4;16:381. doi: 10.1186/s12885-016-2444-5 (PMC4942908; doi:10.1186/s12885-016-2444-5)
Supplement: Supplementary file 1 — Supplementary material. Table S1. Interval between rheumatic disease and cancer diagnosis in years. Table S2. Cancer treatment according to rheumatic diseases. (DOCX 26 kb) [file 12885_2016_2444_MOESM1_ESM.docx]

**Supplementary material**

**Supplementary Table 1. Interval between rheumatic disease and cancer diagnosis in years.**

|  | RA | SLE | SSc | DM/PM | *P* |
| --- | --- | --- | --- | --- | --- |
| Gastric | 6.4 ± 6.2 | 7.2 ± 4.6 | 18 ± 1.0 | 0.5 ± 1.0 | ***0.011*** |
| Colon | 4.5 ± 5.3 | 14.5 ± 18.9 | 6.0 ± 0.0 | 0.2 ± 0.3 | *0.165* |
| Lung | 4.9 ± 4.6 | N/A | 6.9 ± 6 .1 | 1.2 ± 2.9 | *0.084* |
| Breast | 6.4 ± 6.1 | 9.2 ± 8.0 | N/A | 5.0 ± 4.4 | *0.570* |
| *P* | *0.637* | *0.662* | *0.087* | *0.146* |  |

Data are expressed as the mean ± SD years. P value was generated by ANOVA. DM/PM, dermatomyositis/polymyositis; N/A, not applicable; RA, rheumatoid arthritis, RD, rheumatic disease; SLE, systemic lupus erythematosus; SSc, systemic sclerosis; Yrs, years.

**Supplementary table 2. Cancer treatment according to rheumatic diseases.**
